# The increase of MICA gene A9 allele associated with gastric cancer and less schirrous change

**DOI:** 10.1038/sj.bjc.6601750

**Published:** 2004-03-30

**Authors:** S-S Lo, Y-J Lee, C-W Wu, C-J Liu, J-W Huang, W-Y Lui

**Affiliations:** 1 I-Lan Hospital, DOH, Taipei, Taiwan; 2 Division of General Surgery, Taipei-Veterans General Hospital, National Yang Ming University, No. 201, Sec 2, Shih-pai Road, Taipei, Taiwan; 3 Department of Medical Research and Pediatrics, Mackay Memorial Hospital, Taipei, Taiwan; 4 School of Dentistry, National Yang Ming University, Taiwan

**Keywords:** MICA gene, gastric cancer, polymorphism

## Abstract

Since surgical resection is the principal treatment of gastric cancer, early detection is the only effective strategy against this disease at present. Recently, a new polymorphic gene family, the major histocompatibility complex class I chain-related (MIC) genes located about 40 kb centromeric to HLA-B gene has been proposed. This family consists of five genes (A, B, C, D and E). Among them, MICA has five various alleles (A4, A5, A5.1, A6 and A9), which can be used as a polymorphic marker for genetic mapping and for disease susceptibility. The MICA polymorphism was studied in our gastric cancer patients to see if there is any possible correlation with genetic predisposition and clinicopathological factors. Genomic DNA was extracted from fresh or frozen peripheral blood leukocytes in 107 patients with gastric adenocarcinoma who underwent gastrectomy in our hospital and 351 noncancer controls. MICA polymorphism was analysed by using PCR-based technique. The results showed both phenotypic and allele frequencies of allele A9 in patients with gastric cancer were significantly higher than controls (33 *vs* 17.6%, *P*=0.005; 17 *vs* 9.9%, *P*=0.02). Gastric adenocarcinoma with allele A9 was associated with less schirrous change than those without (*P*=0.014). MICA gene A9 allele might confer the risk of gastric cancer and associate with less schirrous change. The mechanisms among them deserve further investigation.

Although the global incidence of gastric cancer is decreasing, gastric cancer is still one of the leading cancers in most Asian countries. Its current incidence in Taiwan is 15.19 per 100 000. Since surgical resection is the principal treatment, early detection is the only effective strategy against this disease at present. Human leukocyte antigen (HLA) has been reported to be associated with tumour susceptibility (Lee *et al*, 1996b), lymph node metastasis (Ogoshi *et al*, 1996), induction of cytotoxic T-lymphocytes (Nabeta *et al*, 2000) and HER-2/neu overexpression (Kono *et al*, 2002) in patients with gastric adenocarcinoma. However, its application in tumour screening or prognosis remains to be investigated. Recently, a new polymorphic gene family, the major histocompatibility complex (MHC) class I chain-related genes located about 40 kb centromeric to HLA-B gene have been identified (Bahram *et al*, 1994). This family consists of five genes: MHC class I chain-related gene A (MICA), gene B (MICB), gene C (MICC), gene D (MICD) and gene E (MICE). MICC, MICD, MICE are pseudogenes, while MICA and MICB encode proteins that are involved in cellular responses to stress (Bahram and Spies, 1996; Groh *et al*, 1998).

Among them, MICA has a triplet repeat microsatellite polymorphism (GCT)_n_ in the transmembrane region, which consists of five alleles, A4, A5, A5.1, A6 and A9 (Mizuki *etal*, 1997). According to the open reading frame of the MICA cDNA, the microsatellite encodes polyalanine and therefore the number of alanine residues differs by the number of triplet repeats. For example, an A4 is defined to contain four GCT repeats and A5.1 contains five triplet repeats plus one additional nucleotide insertion (GCCT) causing a frameshift mutation. The alleles vary among individuals, and hence polymorphism of MICA can be used for genetic mapping and analyses of disease susceptibility. For example, increased frequency of MICA A6 allele was found in patients with oral squamous cell carcinoma (Liu *et al*, 2002), Behcet's disease (Molinotti *et al*, 2001), and ulcerative colitis (Sugimura *et al*, 2001). In addition, increased frequency of A9 allele was reported in psoriatic arthritis (Gonzalez *et al*, 2001) and type I diabetes (Lee *et al*, 2000).

We investigated the MICA polymorphism associated with gastric cancer patients in Taiwan in order to see if there is possible correlation with genetic predisposition and clinicopathological factors.

## PATIENTS AND METHODS

### Subjects

In all, 107 consecutive gastric cancer patients who underwent gastrectomy in Taipei-VGH were enrolled into this study and their clinicopathological factors were recorded according to our prospective database. A total of 351 control subjects were selected from people who came for routine physical check up. Those with autoimmune disorders, blood disease and previous malignancy were excluded. After an informed consent was obtained, blood was drawn from the subjects to extract genomic DNA.

### Polymorphysim analysis

A PCR-based polymorphism analysis was used in this study. Genomic DNA was extracted from fresh or frozen peripheral blood leukocytes by standard technique (Buffone and Darlington, 1985; Lee *et al*, 1996a). Primers (MICA5F, 5′-CCTTTTTTTCAGGGAAAGTGC-3′ and MICA5R, 5′-CCTTACCATCTCCAGAAACTGC-3′) flanking the transmembrane region were designed based on the reported sequence (Bahram *et al*, 1994; Ota *et al*, 1997). The MICA5F primer corresponds to the intron 4 and exon 5 boundary regions, and MICA5R is located in intron 5 (Ota *et al*, 1997). MICA5R was 5′ end-labelled with fluorescent dye (Applied Biosystems, Foster City, CA, USA) (Goto *et al*, 1997,^1998^). The amplification reaction mixture (15 *μ*l) contained 50 ng genomic DNA, 10 mM Tris-HCl (pH 9.0), 50 mM KCl, 1.5 mM MgCl_2_, 0.01% gelatin, 0.1% Triton X-100, 0.2 mM of each dNTP, 0.5 *μ*M of each primer and 0.5 U Prozyme DNA polymerase (Protech Enterprise, Taipei, Taiwan). A GeneAmp PCR system (Perkin-Elmer Corporation, Foster City, CA, USA) was used to do the PCR reaction. The reaction mixture was denatured at 95°C for 5 min followed by 10 cycles at 94°C for 15 s, 55°C for 15 s, 72°C for 30 s, then by an additional 20 cycles at 89°C for 15 s, 55°C for 15 s, 72°C for 30 s, and by a final extension at 72°C for 10 min.

Then the PCR products were denatured for 5 min at 100°C, mixed with formamide-containing stop buffer, and subjected to electrophoresis on 4% polyacrylamide gel containing 8-M urea in an ABI Prism 377-18 DNA sequencer (Applied Biosystem). The number of microsatellite repeats was estimated automatically with Genescan 672 software (Applied Biosystem) with a standard size marker of GS-350 TAMRA (*N*,*N*,*N*,*N*-tetramethyl-6-carbonhydroxyl rhodamine, Applied Biosystems) (Goto *et al*, 1997). Alleles were designed according to the classification of Mizuki *et al* (Ota *et al*, 1997). Their amplified sizes are 179 bp (A4), 182 bp (A5), 183 bp (A5.1), 185 bp (A6) and 194 bp (A9). At least two independent experiments were performed on each sample to assure the analyses were reproducible.

### Analyses with clinicopathological factors

Any possible significant alteration of MICA allele will be analysed with their clinicopathological factors, which are based on Japanese criteria (Japanese Gastric Cancer Association, 1998) and include age, sex, tumour location, tumour size, cellular differentiation, gross appearance, histological patterns, stromal reaction (cancer–stroma relationship), depth of invasion, lymph node status and tumour stage to see if there is correlation among them. Based on the amount of stromal tissue, stromal reaction (cancer–stroma relationship) of gastric cancer was classified into scirrhous, medullary and intermediate types by observation of H&E stained pathological sections (Japanese Gastric Cancer Association, 1998). In this study the scirrhous type was quantitatively defined as tumour stroma occupied more than 50% of tumour area, less than 10% in medullary type and 10–50% in the intermediate type. The three categories were determined under × 40 (low power field) magnification field.

### Statistical analysis

The difference of phenotype and gene frequencies between patients and normal controls were analysed by using *χ*^2^ test. Significant alteration of MICA allele also was analysed with clinicaopathological factors by using *χ*^2^ test. Statistically significant difference was defined as *P*<0.05.

## RESULTS

To establish the phenotypic frequencies of MICA alleles in Taiwanese population, we have analysed 351 normal samples. The controls' ages ranged from 22 to 71 years (mean+s.d.=42.1+10.7). Age did not affect the MICA alleles distribution. The gender distribution of the control was 185 : 166, male to female. The analyses concluded that A4 is 31%, A5 is 50%, A5.1 is 36%, A6 is 8% and A9 is 18%. This is important for this study, since the frequencies are different in various areas (Table 1

**Table 1:** Phenotypic frequencies of MICA alleles in various countries

**Allele**	**Taiwan^a^ (*n***=**351) (%)**	**Japan^b^ (103) (%)**	**Spain^b^ (342) (%)**	**Sweden^b^ (153) (%)**
A4	31	30	23	26
A5	50	52	21	56
A5.1	36	17	43	66
A6	8	46	55	9
A9	18	31	29	18

a Current study.

b Petersdorf *et al* (1999).

). With this information available to us, we then analysed and compared samples from 107 patients with gastric adenocarcinoma who underwent gastrectomy in our hospital. Although no significant difference of frequency of A4, A5, A5.1 and A6 alleles was found between normal controls and gastric cancer patients, both the phenotypic and allelic frequencies of A9 were significantly higher than those in normal controls (33 *vs* 17.6%, *P*=0.005; 17 *vs* 9.9%, *P*=0.02) (Tables 2

**Table 2:** Phenotype frequencies of MICA gene in gastric carcinoma patient and normal control

	**Normal**		**Gastric ca**			
**Phenotype**	**Number (*N*=351)**	**Frequency**	**Number (*N*=107)**	**Frequency**	***P***-**value**	**Corrected *P***
A4	107	30.4%	35	33%	0.663	3.315
A5	174	49.5	59	55	0.313	1.565
A5.1	127	36.1	40	37	0.821	4.105
A6	27	7.6	13	12	0.153	0.765
A9	62	17.6	35	33	0.001^a^	0.005^a^

a Statistic significance.

and 3

**Table 3:** Allelic frequencies of MICA gene in gastric carcinoma patient and normal control

	**Normal**		**Gastric ca**			
**Allele**	**Number (*N*=702)**	**Frequency**	**Number (*N*=214)**	**Frequency**	***P***-**value**	**Corrected *P***
A4	161	22.9%	41	19%	0.244	1.22
A5	262	37.3	73	34	0.393	1.965
A5.1	177	25.2	49	23	0.491	2.455
A6	32	4.5	14	7	0.245	1.225
A9	70	9.9	37	17	0.004^a^	0.02^a^

a Statistic significance.

).

We further examined whether the A9 allele might contribute to the clinicopathological factors of these patients. Several clinicopathological factors such as age, sex, tumour location, size, gross appearance, histological patterns, depth of invasion, lymph node status and TNM staging were included in the analyses. Among these factors, we found that gastric cancer with allele A9 was strongly associated with less schirrous reaction (stromal reaction) compared with ‘non-A9’ gastric tumours (*P*=0.014) (Table 4

**Table 4:** Clinicopathological features of gastric cancer with A9 phenotype MICA

	**MICA phenotype**		
**Parameter**	**A9 (*n*=35)**	**Non-A9 (*n*=72)**	***P***-**value**
*Age*			
<65	15	26	0.617
>65	20	46	
			
*Sex*			
Male	26	55	0.978
Female	9	17	
			
*Tumour location*			
Upper 1/3	7	9	0.512
Middle 1/3	10	28	
Lower 1/3	18	34	
Whole	0	1	
			
*Tumour size*			
<4 cm	19	30	0.287
4–8 cm	12	35	
>8 cm	4	7	
			
*Cell differentiation*			
Well	1	1	0.424
Moderate	19	30	
Poor	15	41	
			
*Borrmann type*			
0	19	30	0.256
I+II	5	11	
III+IV	11	31	
			
*Infiltration type*			
Alpha	12	24	0.466
Beta	12	18	
Gamma	11	31	
			
*Stromal reaction*			
Medullary	9	23	0.014^a^
Intermediate	25	33	
Schirrous	1	16	
			
*Ming classification*			
Infiltrative	15	26	0.560
Expanding	20	46	
			
*Lauren's classification*			
Intestinal type	22	36	0.191
Diffuse type	13	36	
			
*Depth of tumour invasion*			
T1	14	28	0.744
T2	6	10	
T3+T4	15	34	
			
*Lymph node metastasis*			
Negative	23	36	0.123
Positive	12	36	
			
*Liver metastasis*			
Negative	33	71	0.221
Positive	2	1	
			
*Peritoneal dissemination*			
Negative	34	70	1.000
Positive	1	2	
			
*TNM stage*			
I	18	34	0.292
II	7	6	
III	6	21	
IV	4	11	

a Statistic significance.

), suggesting that gastric cancer patients with allele A9 associated with less schirrous reaction.

## DISCUSSION

In this study, we have shown that the MICA allele A9 was significantly correlated with gastric adenocarcinoma and less schirrous change in gastric cancer tissue. These findings suggest that MICA allele A9 may be important in the etiology and immune reaction of gastric adenocarcinoma. Although status of stromal reaction is not routinely included in pathological report, it was reported to be a prognostic indicator of gastric cancer (Wu *et al*, 1997). Stromal reaction of tumour was shown to relate with cancer desmoplastic reaction (Ohtani *et al*, 1992), angiogenesis (Engels *et al*, 1997), tumour invasion and metastasis, tumour cell proliferation (Wernet, 1997), and immune reactions (Saiki *et al*, 1996). Tumour stroma is composed of new blood vessels, inflammatory cells and connective tissue (Dvorak, 1986). Tumour stromal reaction include many complicated interrelated processes, including production of cytokine (interleukine) to induce immune T cells to elicit tumour regression (Nabeta *et al*, 2000), expressing adhesion molecules and production of matrix-degrading enzyme by stromal cells to facilitate tumour invasion and metastasis (Wernet, 1997). Currently, the cellular and molecular events of stromal reaction were proposed to be similar to those of wound healing (Dvorak, 1986) and inflammatory diseases, such as ulcerative disease and Crohn's disease (Ohtani, 1998). In these inflammatory lesions, the aberration of the immune system is speculated to be the cause of the diseases. Stromal reaction of tumour can also be regarded as an immune response to a neogrowth. Therefore, the host immune reactions can be regarded as a factor in modulating the aggressiveness of a tumour. For desmoplastic reaction, it is still uncertain whether it is defensive for the host or it is facilitating the tumour growth, although a poorer survival was reported in patients with gastric cancer and breast cancer (Cardone *et al*, 1997; Caporale *et al*, 2001). As shown in the current study, less scirrhous (less desmoplastic reaction) type tumour appeared in A9 allele group (*P*=0.014) and was probably resulted from some host immune mechanism.

MICA encodes a molecule similar to MHC class I antigens and may share the same capacity of binding to short peptides or small ligands. MICA is expressed in fibroblasts, epithelial cells (Bahram *et al*, 1994), keratinocytes, endothelial cells and monocytes (Zwirner *et al*, 1998), and may play a role in the immune response (Bahram *et al*, 1994). Its expression is regulated by a promoter heat shock element similar to those of heat shock protein (HSP) genes (Groh *et al*, 1996). High levels of MICA expression in epithelial cell lines together with upregulation of MICA after heat shock may represent a new molecular mechanism of exposing stressed epithelial cells to the immune system (Bahram *et al*, 1996). It is shown that HSPs are involved in the formation of malignancy (Bonay *et al*, 1994; Kawanishi *et al*, 1999), including gastric adenocarcinoma (Liu *et al*, 1999; Maehara *et al*, 2000). In addition, they are expressed by transformed/cancer cells, which are important targets for T lymphocytes. High levels of MICA expression in epithelial cells after heat shock (stress) may not be coincident. It may provide a mechanism of exposing transformed cells to the mucosal immune system allowing *γδ* T cells (Bahram *et al*, 1996; Groh *et al*, 1998), a subset of T cells expressing the *γδ* T cell receptors (TCRs) *γ*/*δ* heterodimer (Haas *et al*, 1993), to recognise and destroy transformed/damaged cells. Although *γδ* T cells constitute only about 5% of circulating T cells, they are distributed throughout the human intestinal epitheliaum and may function as sentinels that respond to self-antigens. Interestingly, MICA is almost exclusively expressed intestinal epitheliaum. Recently, it was shown that NK cells and antigen-specific effector T cells could be triggered by MIC engagement of NKG2D (Bauer *et al*, 1999), a receptor expressed on most NK cells, *γδ* T cells and CD8 *γδ* T cells involved in the innate and adaptive immune responses (Bauer *et al*, 1999). However, circulatory MICA secreted by neoplasms can downregulate the expression of NKG2D and impair the responsiveness of effector T cells (Groh *et al*, 2001). Whether MICA A9 antigen product can result in altered immunity and susceptibility to gastric cancer via reactions with HSPs or *γδ* T cell or NKG2D need further investigation (Groh *et al*, 1998,^2001^,^2002^; Bauer *et al*, 1999).

In summary, all of the above findings suggesting MICA may relate with host immunity. Since its alleles vary among individuals and may confer variable disease susceptibility, analyses of MICA alleles maybe useful in cancer investigation. Our results demonstrated that Taiwanese carrying an A9 allele have higher risk to gastric cancer. Furthermore, gastric cancers with A9 allele are associated with less schirrous change. Further investigation can study the mechanism of activity of MICA A9 allele. Identification of the mechanism of association of MICA A9 allele with gastric cancer could help the individuals most likely benefit from cancer screening and prevention program and may suggest novel treatment modality.
